# Emodin relieves the inflammation and pyroptosis of lipopolysaccharide-treated 1321N1 cells by regulating methyltransferase-like 3 -mediated NLR family pyrin domain containing 3 expression

**DOI:** 10.1080/21655979.2022.2045836

**Published:** 2022-03-04

**Authors:** Bu Wang, Yuan Liu, Rui Jiang, Zhiliang Liu, Haiyun Gao, Fenqiao Chen, Jianqiang Mei

**Affiliations:** a Department of Emergency, The First Affiliated Hospital of Hebei Traditional Chinese Medicine University, Shijiazhuang, Hebei, China; b Department of Emergency Critical Care Medicine, East Branch of the Second Hospital of Hebei Medical University, Shijiazhuang, Hebei, China; c Department of Critical Care Medicine, The First Affiliated Hospital of Hebei Traditional Chinese Medicine University, Shijiazhuang, Hebei, China; d Department of Basic Nursing, School of Nursing, Hebei Medical University, Shijiazhuang, Hebei, China; e Department of Emergency, Hebei Yiling Hospital, Shijiazhuang, Hebei, China

**Keywords:** Sepsis, emodin, pyroptosis, inflammation, NLRP3

## Abstract

Sepsis brain injury (SBI) is a major cause of death in critically ill patients. The present study aimed to investigate the role of emodin in SBI development. Human astrocyte 1321N1 cells were stimulated with 100 ng/mL lipopolysaccharide (LPS) to establish an SBI model in vitro. Flow cytometry was performed to measure the cell pyroptosis. The protein expression levels of syndecan-1 (SDC-1), NLR family pyrin domain containing 3 (NLRP3), Caspase-1, and the N-terminal fragment of gasdermin D (GSDMD-N) were measured using Western blotting. Interleukin (IL)-1β, IL-6, IL-10, and tumor necrosis factor (TNF)-α levels in cells were measured using enzyme-linked immunosorbent assay kits. The N^6^-methyladenosine (m^6^A) modification was analyzed using the methylated RNA immunoprecipitation assay. NLRP3 activator, nigericin, was used to overexpress NLRP3. LPS treatment significantly enhanced the pyroptosis in 1321N1 cells, increased the levels of TNF-α, IL-1β, and IL-6, and decreased the levels of IL-10. The protein expression levels of NLRP3, SDC-1, GSDMD-N, and Caspase-1 were also increased. Emodin treatment decreased the levels of TNF-α, IL-1β, IL-6, NLRP3, SDC-1, GSDMD-N, and Caspase-1, while increasing the levels of IL-10 in LPS-treated 1321N1 cells. Nigericin reversed the effects of emodin. Furthermore, emodin upregulated m^6^A levels in NLRP3 by increasing the expression of methyltransferase-like 3 (METTL3). Meanwhile, knockdown of METTL3 reversed the effects of emodin on the mRNA expression and stability of NLRP3. Therefore, emodin inhibits the inflammation and pyroptosis of LPS-treated 1321N1 cells by inactivating METTL3-mediated NLRP3 expression.

## Graphical abstract

**Figure:**
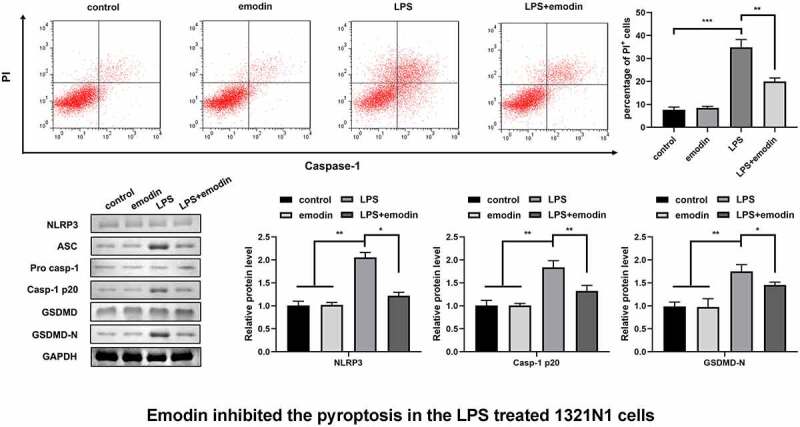

## Introduction

Sepsis is a major cause of death in critically ill patients. Clinically, it results in multisystem complications, including brain injury, infection, and stress [1]. Sepsis is also associated with the abnormal activation of various immune-related signaling pathways by many epidemiological factors [2]. Although the research on sepsis is rapid, the incidence of sepsis remains high [3,4]. Studies have demonstrated that immune dysfunction, including excessive inflammatory response and immunosuppression, is a vital pathophysiological mechanism in sepsis [5]. Worsening peripheral inflammation can lead to neuroinflammation, which further causes serious damage to the central nervous system. Subsequently, the destruction of the blood-brain barrier related to sepsis promotes the activation of glial cells and triggers a storm of proinflammatory cytokines in the central nervous system, resulting in brain dysfunction in the survivors [6]. Despite the investment of considerable time and energy, the treatment of septic brain injury still lacks successful interventions. Recent studies have shown that pyroptosis of peripheral blood immune cells can occur in patients with sepsis [7,8]. Pyroptosis is a type of programmed cell death process. The inflammatory reaction plays an important role in sepsis progression, and the unique inflammatory characteristics of pyroptosis suggest that it may affect sepsis by participating in inflammatory reactions [9]. Therefore, pyroptosis may be an important factor in sepsis-induced brain injury therapy.

Emodin is the main active component of rhubarb. In recent years, studies have confirmed that rhubarb has pharmacological activities, such as anti-inflammatory, antibacterial, antiviral, antioxidant, and immune regulation activities, and it also improves the microcirculation [10,11]. Emodin has gradually become a research hotspot for severe inflammatory diseases, such as sepsis, due to its significant anti-inflammatory effects. In recent years, many studies have reported that emodin exhibits significant neuroprotective activity. At present, the neuroprotective activity of emodin is considered to be related to the inhibition of systemic inflammation and neuroinflammatory responses in the brain, improving tissue metabolic disorders, and regulating the acetylcholine system [^12–14^]. The neuroprotective effect of emodin has been confirmed in many animal models, such as the traumatic brain injury, cerebral ischemia-reperfusion brain injury, hypoxic brain injury, and focal cerebral ischemia injury models [^15–19^]. However, there are few reports on the neuroprotective effects of emodin in a lipopolysaccharide (LPS)-induced sepsis brain injury (SBI) model.

Hence, the objective of the current study was to explore the effects of emodin on LPS-induced septic brain injury. We hypothesized that emodin inhibits pyroptosis and inflammation in LPS-treated 1321N1 cells by regulating N^6^-methyladenosine (m^6^A)-mediated NLR family pyrin domain containing 3 (NLRP3) expression.

## Materials and methods

### Cell culture and treatment

Human astrocytoma 1321N1 cells were obtained from the cell bank of the Shanghai Chinese Academy of Sciences (Shanghai, China) and cultured in Dulbecco’s modified Eagle’s medium (SenBeiJia, Nanjing, China) supplemented with 10% fetal bovine serum and 100 U/mL penicillin-streptomycin (SenBeiJia). The cells were then incubated with 100 ng/mL LPS for 24 h to establish a sepsis model. Subsequently, the cells were divided into control, LPS, 20 μM emodin, LPS+emodin, and LPS+emodin+20 μM nigericin groups. All cells were cultured in a 5% CO_2_ incubator at 37°C for 24 h.

### Cell transfection

Cells in the log growth phase were transfected with overexpressed methyltransferase-like 3 (METTL3), negative control vector (sh-METTL3), and negative control shRNA (Sangon, China) using Lipofectamine 3000 reagent (Invitrogen, CA, USA) according to a previous study [20].

### Flow cytometry

Cell death was detected using FAM FLICATM Caspase-1 Kit (Bio Rad, USA) by flow cytometry according to a previous study [21]. The cells in each group were placed in a fluorescence-activated cell sorting tube and resuspended. The cells were then treated with 1 µg/mL PI and caspase-1, then cultured in the dark for 5 min. Finally, PI fluorescence was measured using a flow cytometer (Agilent Technologies, USA).

### Western blotting

The Western blot assay was performed according to previous study [22]. Primary antibodies against NLRP3 (1:800), apoptosis-associated speck-like protein (ASC; 1:1200), Caspase-1 (1:600), gasdermin D (GSDMD; 1:1500), N-terminal fragment of gasdermin D (GSDMD-N; 1:1000), syndecan-1 (SDC-1; 1:1000), and glyceraldehyde-3-phosphate dehydrogenase (GAPDH; 1: 2000) were purchased from Abcam (Cambridge, UK). Radioimmunoprecipitation assay (Beyotime, China) reagent was used to separate proteins from the cells. The total protein concentration was measured using a BCA Protein Assay Kit (Beyotime). Subsequently, all proteins were separated by 10% sodium dodecyl sulfate-polyacrylamide gel electrophoresis and electrotransferred to polyvinylidene fluoride membranes. Next, 5% skim milk was used to block the membranes for 60 min and the membranes were supplied with primary antibodies and incubated at 4°C for 12 h. Next day, the membranes were incubated with secondary antibodies (1: 2000, Abcam) for 120 min. Finally, protein expression was determined using an ECL kit (Beyotime) and the Scion Image v. 4.0.2 software.

### Determination of cytokine levels

According to a previous study [23], the levels of interleukin (IL)-1β, IL-6, IL-10, and tumor necrosis factor (TNF)-α were determined using the enzyme-linked immunosorbent assay kits provided by EiAab Technology Co., Ltd (Wuhan, China). All procedures were performed strictly according to the requirements of the kit.

### Methylated RNA immunoprecipitation (MeRIP) assay

Forty micrograms of total RNA was extracted for the MeRIP assay according to a previous study [24]. Purified mRNA fragments were incubated with an m^6^A antibody for immunoprecipitation using a MeRIP m6A kit (Ribobio, Guangzhou, China). All operations were performed strictly in accordance with the manufacturer’s instructions. The final enriched m^6^A-modified RNA was verified and analyzed using reverse transcription-quantitative polymerase chain reaction (RT-qPCR).

### RT-qPCR

RNA was extracted using the TRIzol reagent (Beyotime, Nantong, China). RNA was reverse-transcribed to cDNA using a reverse transcription kit (Takara, Dalian, China). All primers were purchased from Sangon Biotechnology Technology Co. Ltd. (Shanghai, China). RT-qPCR was conducted on a CFX96 Real-Time PCR Detection System (BioFortune Sciences Co., Ltd.) using a RT-qPCR Kit (Takara). The reaction conditions were as follows: 95°C, 2 min; 95°C, 30s; 53°C, 1 min; 72°C, 30s; 35 cycles; 72°C, 10 min. Results were calculated using the 2^−ΔΔCt^ methods. *GAPDH* was used as the housekeeping gene.

### Statistical analysis

Statistical analyses were conducted using SPSS 20.0 (IBM, SPSS, USA). The results of this study are presented as the mean ± standard deviation (SD). One-way analysis of variance followed by Duncan’s post-hoc test was used for the analysis of differences among multiple groups. P values < 0.05 were considered to be statistically significant.

## Results

This study confirmed that emodin relieves pyroptosis, inflammation, and glycocalyx dysfunction in LPS-treated 1321N1 cells. In addition, emodin upregulated METTL3 expression levels and promoted the m6A methylation modification-induced downregulation of NLRP3 in LPS-treated 1321N1 cells. Emodin inhibits the inflammation and pyroptosis of LPS-treated 1321N1 cells by inactivating METTL3-mediated NLRP3 expression.

### LPS promoted the pyroptosis of 1321N1 cells

**Figure 1.**
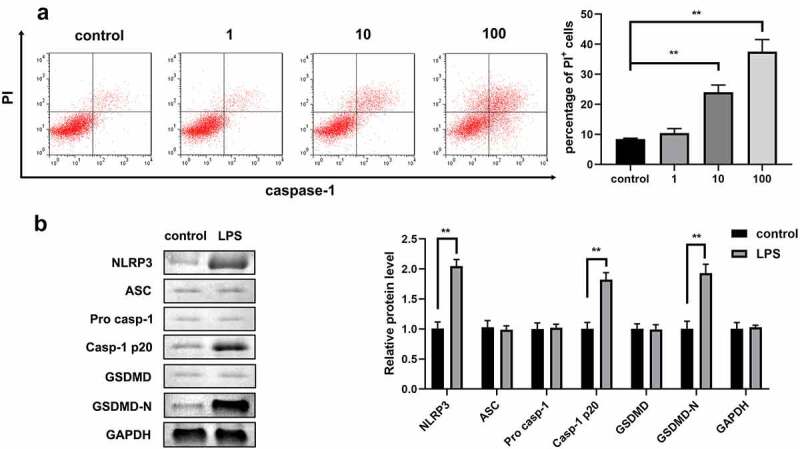
Lipopolysaccharides (LPSs) promoted the pyroptosis of 1321N1 cells. (a) Dead 1321N1 cells treated with LPS (1, 10, and 100 ng/mL) were analyzed using propidium iodide (PI) staining. **P < 0.01. (b) Protein expression levels of NLR family pyrin domain containing 3 (NLRP3), apoptosis-associated speck-like protein (ASC), pro-caspase-1, caspase-1-p20, gasdermin D (GSDMD), and N-terminal fragment of gasdermin D (GSDMD-N) in LPS (100 ng/mL)-treated 1321N1 cells were measured using Western blotting. **P < 0.01.

As shown in Figure 1(a), after treatment with different doses of LPS, the number of PI-positive cells dramatically increased in a dose-dependent manner. Hence, 100 ng/mL LPS was used to carry out further experiments. Next, we detected the protein expression of autophagy-related genes and confirmed that the protein levels of NLRP3, Caspase-1, and GSDMD-N were significantly upregulated after treatment with 100 ng/mL LPS (Figure 1(b)).

### Emodin inhibited the pyroptosis of LPS-treated 1321N1 cells

**Figure 2.**
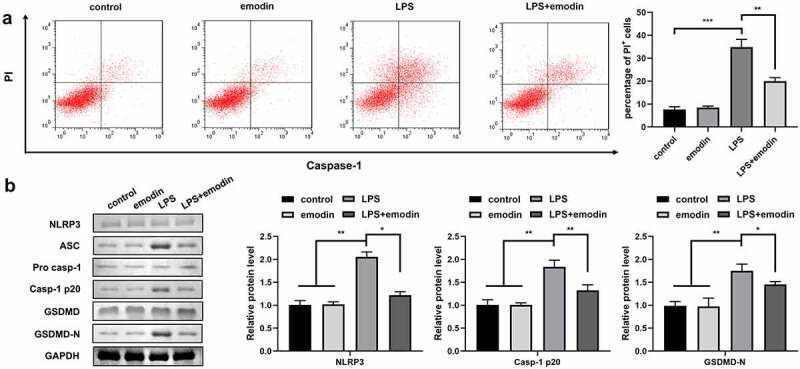
Emodin inhibited the pyroptosis of LPS-treated 1321N1 cells. (a) After 20 μM emodin and 100 ng/ml LPS treatment, the dead 1321N1 cells were analyzed using PI staining. **P < 0.01, ***P < 0.001. (b) After 20 μM emodin and 100 ng/ml LPS treatment, the protein levels of NLRP3, caspase-1-p20, and GSDMD-N in 1321N1 cells were determined using Western blotting. *P < 0.05, **P < 0.01.

We explored the effect of emodin on autophagy in LPS-treated 1321N1 cells. After emodin pretreatment, the number of PI-positive cells dramatically decreased in LPS-treated 1321N1 cells (Figure 2(a)). In addition, emodin treatment dramatically downregulated the protein levels of NLRP3, Caspase-1, and GSDMD-N in LPS-treated 1321N1 cells, and emodin did not affect 1321N1 cells (Figure 2(b)).

### Emodin relieved the cytokines levels and SDC-1 protein levels in LPS-treated 1321N1 cells

**Figure 3.**
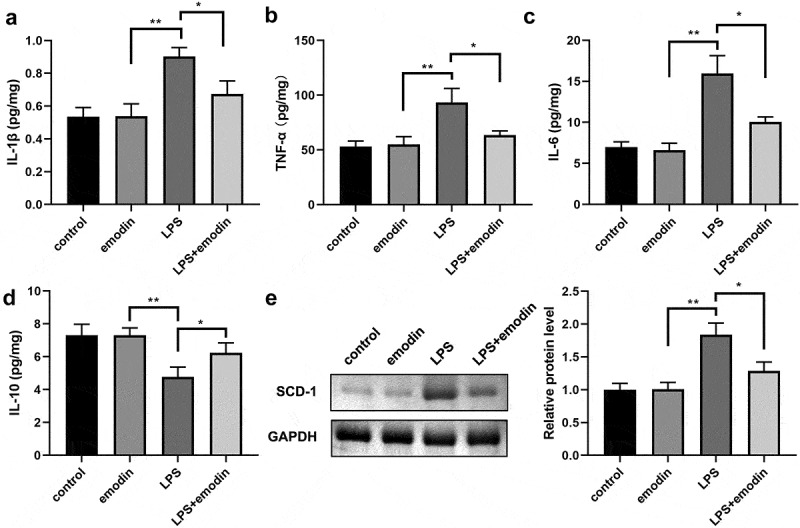
Emodin relieved the inflammation in LPS-treated 1321N1 cells. (a-d) After 20 μM emodin treatment, interleukin (IL)-1β, IL-6, IL-10, and tumor necrosis factor (TNF)-α levels in LPS (100 ng/mL)-treated 1321N1 cells were analyzed. *P < 0.05, **P < 0.01. (e-f) After 20 μM emodin treatment, SDC-1 protein expression levels in LPS (100 ng/mL)-treated 1321N1 cells were determined using Western blotting. *P < 0.05, **P < 0.01.

Subsequently, we analyzed the effect of emodin on the levels of IL-1β, TNF-α, IL-6, and IL-10 in LPS-treated 1321N1 cells. After LPS treatment, IL-1β, TNF-α, and IL-6 levels were dramatically upregulated, IL-10 levels were dramatically downregulated, and emodin significantly decreased IL-1β, TNF-α, and IL-6 levels and increased IL-6 levels in LPS-treated 1321N1 cells. Meanwhile, emodin showed no effect on 1321N1 cells (Figure 3(a-d)). Next, we explored the role of emodin on SDC-1 expression. We found that SDC-1 protein expression was upregulated in the LPS group and was downregulated in the LPS+emodin group. Emodin had no effect on 1321N1 cells (Figure 3(e)).

### Nigericin reversed the effect of emodin in LPS-treated 1321N1 cells

**Figure 4.**
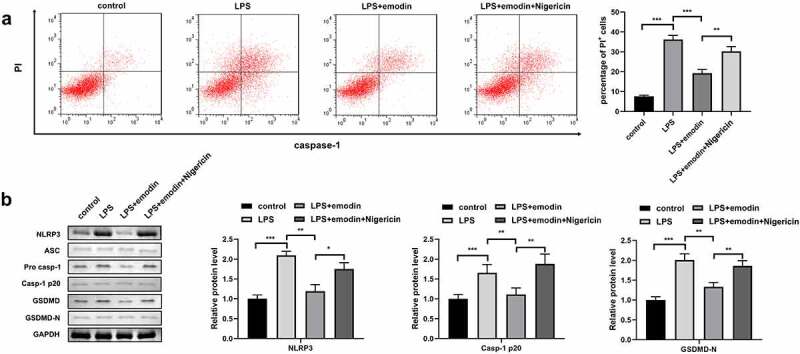
Nigericin reversed the effects of emodin on the pyroptosis of LPS-treated 1321N1 cells. (a) After 20 μM emodin and 20 μM nigericin treatment, dead LPS (100 ng/mL)-treated 1321N1 cells were analyzed using PI staining. **P < 0.01, ***P < 0.001. (b) After 20 μM emodin and 20 μM nigericin treatment, the protein levels of NLRP3, caspase-1-p20, and GSDMD-N in LPS (100 ng/mL)-treated 1321N1 cells were determined using Western blotting. *P < 0.05, **P < 0.01. *P < 0.05, **P < 0.01, ***P < 0.001.

**Figure 5.**
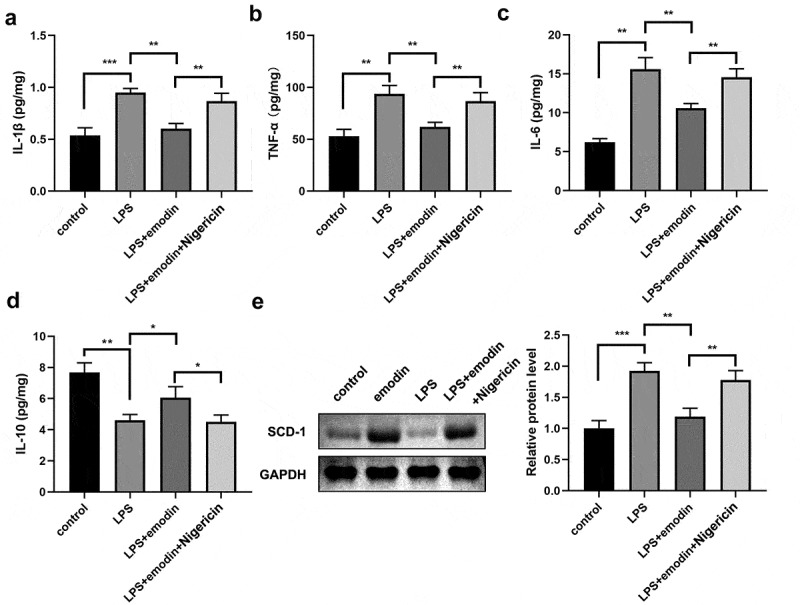
Nigericin reversed the effects of emodin on the cytokines levels and SDC-1 protein levels in LPS-treated 1321N1 cells. (a-d) After 20 μM emodin and 20 μM nigericin treatment, IL-1β, TNF-α, IL-6, and IL-10 levels in LPS (100 ng/mL)-treated 1321N1 cells were analyzed. *P < 0.05, **P < 0.01, ***P < 0.001. (e-f) After 20 μM emodin and 20 μM nigericin treatment, SDC-1 protein expression levels in LPS (100 ng/mL)-treated 1321N1 cells were determined by Western blotting. **P < 0.01, ***P < 0.001.

Next, the 1321N1 cells were incubated with the NLRP3 activator, nigericin. We found that nigericin antagonized the effects of emodin on PI-positive cells and protein levels of NLRP3, Caspase-1, and GSDMD-N in LPS-treated 1321N1 cells (Figure 4(a,b)). In addition, we also found that nigericin reversed the effects of emodin on IL-1β, TNF-α, IL-6, and IL-10 levels and SDC-1 protein expression levels in LPS-treated 1321N1 cells (Figure 5(a-e)).

### Emodin downregulated NLRP3 expression by increasing the m^6^A RNA methylation regulator, METTL3, in LPS-treated 1321N1 cells

**Figure 6.**
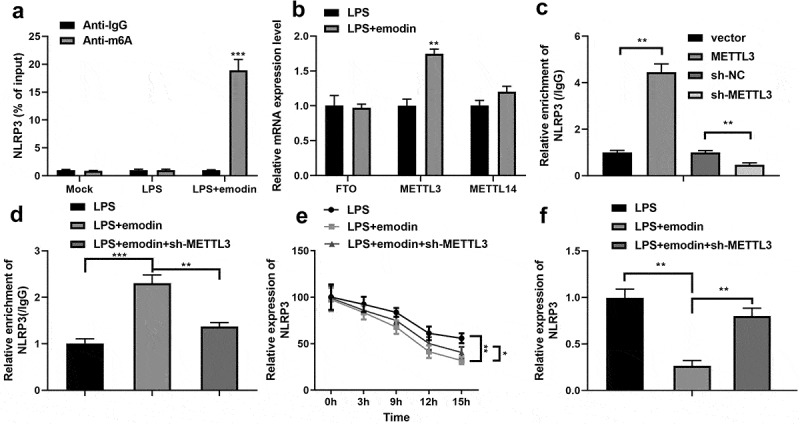
Emodin downregulated NLRP3 expression by increasing the N^6^-methyladenosine (m^6^A) RNA methylation regulator, methyltransferase-like 3 (METTL3), in LPS-treated 1321N1 cells. (a) After treatment with 20 μM emodin, m^6^A levels in LPS (100 ng/mL)-treated 1321N1 cells were detected. ***P < 0.001. (b) After 20 μM emodin treatment, mRNA expression levels of the fat mass and obesity-associated protein (FTO), METTL3, and METTL14 in LPS (100 ng/mL)-treated 1321N1 cells were analyzed using reverse transcription-quantitative polymerase chain reaction (RT-qPCR). **P < 0.01. (c) After METTL3 and sh-METTL3 transfection, NLRP3 m^6^A methylation levels were determined. **P < 0.01. (d) After 20 μM emodin and sh-METTL3 treatment, NLRP3 m^6^A methylation levels in LPS (100 ng/mL)-treated 1321N1 cells were measured. **P < 0.01, ***P < 0.001. (e) After 20 μM emodin and sh-METTL3 treatment, the mRNA stability of NLRP3 in LPS (100 ng/mL)-treated 1321N1 cells was analyzed using RT-qPCR. *P < 0.05, **P < 0.01. (f) After 20 μM emodin and sh-METTL3 treatment, mRNA expression levels of NLRP3 in LPS (100 ng/mL)-treated 1321N1 cells were measured using RT-qPCR. **P < 0.01.

Finally, we found that emodin dramatically increased the m6A levels of NLRP3 in LPS-treated 1321N1 cells (Figure 6(a)). In addition, RT-qPCR results showed that emodin dramatically upregulated the mRNA expression of METTL3 in LPS-treated 1321N1 cells (Figure 6(b)). Furthermore, after sh-METTL3 or overexpressed-METTL3 transfection, NLRP3 m6A methylation levels were significantly downregulated or upregulated in 1321N1 cells (Figure 6(c)), respectively. After sh-METTL3 transfection, the effect of emodin on the levels of m6A methylation and mRNA and protein expression of NLRP3 was antagonized (Figure 6(d-f)). Furthermore, knockdown of METTL3 reversed the emodin-induced decrease in the mRNA stability of NLRP3 (Figure 6(g)).

## Discussion

In the current study, we demonstrated that emodin plays a protective role against SBI. Emodin suppressed pyroptosis and inflammatory reaction in LPS-treated 1321N1 cells by increasing the m6A level of in NLRP3.

Various signaling molecules are produced in the initial stage of the pathophysiology of sepsis, such as damage-associated molecular patterns (DAMPs) and pathogen-associated molecular patterns (PAMPs), which interact with pattern recognition receptors and recruit immune cells [25]. NLRP3 can identify DAMPs and PAMPs to form a multi-protein complex called the NLRP3 inflammasome, which is involved in the host immune response. The NLRP3 inflammasome includes NLRP3, ASC, and pro-Caspase-1 [26]. NLRP3 interacts with ASC after activation and then recruits pro-Caspase-1 to promote shear activation. Activated Caspase-1 cleaves pro-IL-1β and pro-IL-18 into mature IL-1β and IL-18, respectively, which in turn promotes the inflammatory response [27]. In this study, we found that emodin decreased IL-1β and IL-6 levels, and increased IL-10 levels. In addition, emodin downregulated NLRP3, Caspase-1, and GSDMD-N in LPS-treated 1321N1 cells. Gao et al. [28] demonstrated that emodin effectively relieved acute pancreatitis-associated lung injury by suppressing NLRP3 inflammasome. Similarly, Zhang et al. [29] confirmed that emodin relieved acute pancreatitis damage by inhibiting the NLRP3 pathway. These findings are similar to those of this study. We found that treatment with the NLRP3 activator nigericin reversed the role of emodin in LPS-treated 1321N1 cells. Therefore, we suspected that the NLRP3 inflammasome may be the Achilles’ heel of SBI.

The vascular endothelial glycocalyx is one of the first sites involved in inflammation. Endothelial cell glycocalyx dysfunction can be directly or indirectly manifested in various inflammatory diseases such as diabetes, ischemia reperfusion injury, and sepsis [30]. In sepsis, TNF-α is released by the impairment of endothelial cell barrier function, resulting in abnormal vascular function. This results in microcirculation disorders and systemic circulatory dysfunction throughout the body [31]. Schmidt et al. [32] found that upregulation of TNF-α caused dissociation of the glycocalyx of the pulmonary capillaries. In a previous study, thinning of the glycocalyx on the surface of intestinal myoendothelial cells was detected after LPS injection in rats, indicating damage to the glycocalyx structure [33]. Xiong et al [34]. demonstrated that IL-1β deactivated cAMP-CREB signaling to raise the prospect of preventing sepsis induced inflammatory lung injury by selectively interfering with the cAMP-CREB pathway. Qiu et al [35]. confirmed that IL-6 is a sensitive and specific diagnostic marker for the early diagnosis of neonatal sepsis with PROM. Besides, the role of IL-10 in depressing monocyte function in sepsis was recently demonstrated Park et al [36]. have recently shown that upregulating the IL-10 production have beneficial effects in a mouse model of sepsis. In addition, many reports have confirmed that the serum levels of glycocalyx-related components, such as SDC-1, a marker of glycocalyx damage in endothelial cells, were increased in patients with sepsis. The increase of SDC-1 was positively correlated with the mortality of sepsis [37,38]. This study also demonstrated that emodin decreases SDC-1 protein expression and IL-1β, IL-6 and TNF-α levels, increases the IL-10 levels in LPS-treated 1321N1 cells. These results indicated that emodin may attenuate SBI by relieving glycocalyx dysfunction.

Recently, RNA methylation was shown to be closely related to sepsis development [39,40]. m^6^A methylation is the most common internal modification in mammalian mRNA; it is also a post-transcriptional modification of the internal sequence of eukaryotic mRNA. It was first discovered in bacterial DNA in 1955 [41]. In the process of m^6^A methylation, there are three types of molecules involved: methyltransferase (fat mass and obesity-associated protein, FTO), demethylase (METTL3 and METTL14), and reader (YTH m6A RNA-binding proteins 1–3) [42]. In this study, we found that emodin significantly upregulated NLRP3 m6A methylation and METTL3 expression levels in LPS-treated 1321N1 cells. Knockdown of METTL3 reversed the effects of emodin on m6A modification, mRNA expression, and NLRP3 stability. These results suggest that emodin may decrease NLRP3 expression levels by regulating METTL3 mediated m^6^A methylation.

## Conclusion

In conclusion, this study confirmed that emodin relieves pyroptosis, inflammation, and glycocalyx dysfunction in LPS-treated 1321N1 cells. In addition, emodin upregulated METTL3 expression levels and promoted the m^6^A methylation modification-induced downregulation of NLRP3 in LPS-treated 1321N1 cells.
